# Associations between Age-Related Hearing Loss and Dietary Assessment Using Data from Korean National Health and Nutrition Examination Survey

**DOI:** 10.3390/nu13041230

**Published:** 2021-04-08

**Authors:** Ji Eun Choi, Jungmin Ahn, Il Joon Moon

**Affiliations:** 1 Department of Otorhinolaryngology—Head and Neck Surgery, College of Medicine, Dankook University, Dankook University Hospital, Cheonan 31116, Korea; garimung@gmail.com; 2 Department of Otorhinolaryngology—Head and Neck Surgery, Korea Cancer Center Hospital, Seoul 01812, Korea; jungmin.ahn.0316@gmail.com; 3 Samsung Medical Center, Department of Otorhinolaryngology—Head and Neck Surgery, School of Medicine, Sungkyunkwan University, Seoul 06355, Korea

**Keywords:** hearing loss, presbycusis, vitamin A, nutrition, food, diet

## Abstract

Age-related hearing loss (ARHL) is a major and rapidly growing public health problem that causes disability, social isolation, and socioeconomic cost. Nutritional status is known to cause many aging-related problems, and recent studies have suggested that there are interaction effects between ARHL and dietary factors. We aimed to investigate the association between ARHL and dietary assessment using data from the fifth Korean National Health and Nutrition Examination Survey, which is a nationwide cross-sectional survey that included 5201 participants aged ≥50 years from 2010 to 2012. All participants had normal findings on otoscopic examination and symmetric hearing thresholds of <15 dB between both sides. Nutritional survey data included food consumption and nutrient intake using the 24 h recall method. Data were analyzed using multiple regression models with complex sampling adjusted for confounding factors, such as age, sex, educational level, and history of diabetes. Higher intake of seeds and nuts, fruits, seaweed, and vitamin A were positively associated with better hearing. Our findings suggest that dietary antioxidants or anti-inflammatory food may help reduce ARHL.

## 1. Introduction

Age-related hearing loss (ARHL) is a common cause of hearing loss worldwide, affecting more than half of all adults by age 75 years [1,2,3]. With the rise of an aging global population, the World Health Organization (WHO) estimates that in 2025 there will be more than 500 million individuals who will suffer significant impairment from ARHL [4]. The hallmark of ARHL is progressive bilateral symmetrical sensorineural hearing loss. Hearing loss is mostly marked at higher frequencies, and it usually occurs after age 50 [5,6]. Previous epidemiologic studies have revealed that ARHL negatively affects participation in interpersonal relations, mental health, cognitive function, and quality of life [7,8,9,10,11]. Accordingly, ARHL constitutes an enormous burden from the public health and social perspectives, and it is important to avoid risk factors and identify protective factors to lessen the burden of ARHL on the global aging population [8].

Pathologically, ARH appears to be most related to degeneration of the cochlea, including the hair cells, spiral ganglion cells, and stria vascularis [1,12]. Although the underlying inciting events for ARHL remain unclear, it has been hypothesized that microcirculation abnormalities, systemic inflammation, and formation of free radicals may cause cochlear damage [13,14,15,16]. Multiple factors can influence the onset and severity of ARHL [17]. These factors include environmental (e.g., loud noise exposure, low socioeconomic status), medical (e.g., ototoxic drugs, otologic infections, hypertension, diabetes), genetic components, and hormonal factors (e.g., estrogen) [15,18,19,20]. Dietary factors (e.g., a high-fat diet) may also be associated with an increased risk of developing ARHL [21,22,23]. An animal study demonstrated that a high-fat diet induced mitochondrial damage, oxidative stress, and apoptosis in the spiral ganglion of the cochlea [23].

Recent studies have demonstrated protective effects of dietary antioxidants or anti-inflammatory foods [13,24,25,26]. Vitamins with antioxidant properties may prevent cochlear damage caused by high levels of toxic free radicals produced with aging. Free radical formation of the inner ear is one of the factors associated with ARHL, causing blood flow reduction in the cochlea leading to the death of the hair cells [27,28]. Consistently, ARHL occurs more frequently in patients with mutations in the *N*-acetyl transferase (NAT) gene, which is involved in the metabolism and detoxification of free radicals, than in healthy subjects [29]. Additionally, it is well-known that chronic inflammation observed during aging [30,31] and ARHL severity has been linked to some factors associated with inflammation [16,32]. For example, it has been shown that spiral ganglion cell damage can be induced by the macrophage-mediated immune response [33], while inflammation-associated vascular changes may induce the vasospasm of stria vascularis [19]. Several controlled interventions have demonstrated that inflammatory markers could be decreased by a change in dietary pattern or of single foods [34,35,36]. However, the utility of dietary supplements to preserve hearing remains to be determined. Some prospective studies have not shown any significant effect of dietary vitamins or anti-inflammatory foods in the prevention of ARHL [37,38].

The Korea National Health and Nutrition Examination Survey (KNHANES) may be useful for epidemiologic study to determine the association between dietary factors and ARHL. This survey collects comprehensive information, such as health status, hearing thresholds, and nutritional condition from the general Korean population by using a complex, two-stage clustered, and stratified random sampling method. Two studies have reported on the beneficial effect of dietary vitamins (e.g., vitamin C, riboflavin, niacin, and retinol) on ARHL [39,40]. However, these studies analyzed data using only weights from the older population without complex sample data analysis. With weights alone, estimates are computed as if the measures have been obtained from the number of cases in the entire population rather than the number of cases in the older population. This can lead to biased results because of low variance estimates and cluster effects within the older population [41]. Thus, statistical analysis of national survey data must reflect the complex sample design of the survey by weight adjustment, clustering, and stratification of the sample design. The other study revealed that low-fat and low-protein diet were associated with ARHL using a complex samples logistic regression analysis, but they analyzed only macronutrients such as carbohydrates, proteins, and fats [42]. In addition, three studies did not exclude participants with asymmetric hearing loss, which may occur by other pathologies, such as tumors, infection, or trauma.

The aim of this study was to investigate the association between dietary factors and ARHL using data from the KNHANES with a complex-samples general linear model.

## 2. Materials and Methods

### 2.1. Data Source and Study Population

The datasets used in this study originated from the fifth KNHANES (2010–2012), which is a nationwide cross-sectional survey that has provided health and nutritional status data from South Koreans since 1998. The survey has been conducted annually by the Korea Centers for Disease Control and Prevention (KCDC) and includes a health interview, a nutrition survey, and a health examination survey [43]. Each year, the KNHANES uses a two-stage clustered (region and household) and stratified random sampling method to represent the South Korean population based on its rolling sample design. The KNHANES data and research methodology are officially available on the KNHANES website (https://knhanes.cdc.go.kr accessed on 29 February 2021) on request. All participants in KNHANES provided written informed consent before completing the survey (IRB No. 2010-02CON-21-C, 2011-02CON-06-C, and 2012-01EXP-01-2C).

**Figure 1:**
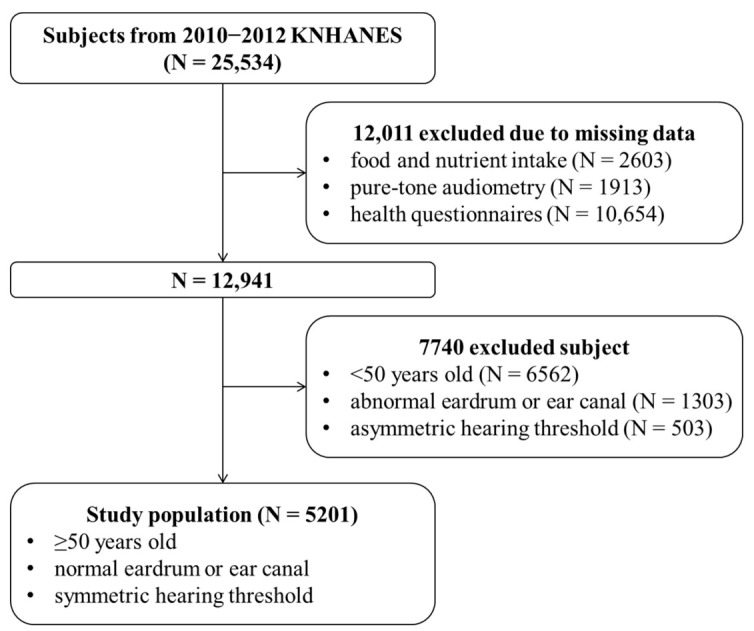
Flow chart of study subject selection.

A total of 25,534 people participated in the fifth KNHANES (8968 in 2010, 8518 in 2011, and 8058 in 2012). Because ARHL is characterized by bilateral symmetrical sensorineural hearing loss and usually occurs after age 50 [5,6], we focused on this study only over 50 years of age and excluded participants who possibility had idiopathic sudden hearing loss, congenital hearing impairment, or conductive hearing loss. The inclusion criteria for this study were: (1) age ≥ 50 years, (2) normal findings on otoscopic examination, and (3) average pure-tone thresholds < 15 dB between both sides at 0.5, 1, 2, and 4 kHz. We excluded participants with missing data on the nutritional survey or for any candidate with risk factors of hearing loss. A total of 5201 participants were included in the final analyses. Figure 1 presents a flow chart of the study population.

### 2.2. Assessment of Hearing and Risk Factors

Participants underwent pure-tone audiometry using supra-auricular headphones in a soundproof booth. Air conduction thresholds were measured at 0.5, 1, 2, 3, 4, and 6 kHz on both ears in accordance with the American National Standards Institute (ANSI). Hearing thresholds were averaged for four frequencies (4FA: 0.5, 1, 2, and 4 kHz) and high frequencies (HF: 4 and 6 kHz).

We selected candidate risk factors that affect hearing impairment: age, sex, income, education level, occupation, occupational noise exposure, current smoking, alcohol consumption, body mass index, and known underlying diseases, such as hypertension and diabetes. Income was equalized into monthly household income and classified into quartiles to determine monthly income level: lower, lower-middle, upper-middle, and upper. Education level was divided into four groups: less than elementary school, middle school, high school, and college or more. Occupational noise exposure history was recorded via a survey question asking if participants had worked in a noisy environment (difficulty in communication with moderate loudness (55 dB HL) between workers) for more than three months. Smoking history was categorized into two groups: never or ex-smoker, and current smoker. Occupation was divided into employed and unemployed groups. Alcohol consumption was divided into two groups according to their drinking behavior: non-drinker and drinker. A non-drinker was defined as someone who had not drank during the last year. Histories of hypertension and diabetes were selected as variables.

### 2.3. Assessment of Dietary Characteristics

Food consumption and nutrient intake were assessed using a 24 h dietary recall method by professional interviewers. Data on all foods consumed by participants over the 24 h prior to the day of the dietary survey were collected. Food items were categorized into 16 food types: (cereals and grain products, potatoes and starches, sugars and sweets, legumes and their products, seeds and nuts, vegetables, mushrooms, fruits, meat and poultry and their products, eggs, fish and shellfish, seaweed, milk and dairy products, oils and fats, beverages, and seasonings). Nutrient intake data included total energy (kcal/day), carbohydrate (g/day), protein (g/day), fat (g/day), crude fiber (g/day), ash (g/day), calcium (mg/day), iron (mg/day), potassium (mg/day), sodium (mg/day), phosphorus (mg/day), retinol (µg/day), β-carotene (µg/day), thiamine (mg/day), riboflavin (mg/day), niacin (mg/day), and Vitamin C (mg/day). Intake of vitamin A (µg retinol equivalents (RE)/day) was calculated by addition of retinol (µg/day) and β-carotene/6 (µg/day).

### 2.4. Statistical Analyses

All statistical analyses were performed using SPSS (version 20.0; IBM Corp., Armonk, NY, USA). As recommended by the Korea Centers for Disease Control and Prevention, this study was performed with a complex sample module including a stratification variable, a clustering variable, and a weight variable. Categorical variables are denoted by unweighted frequencies and weighted percent, and continuous variables are denoted by weighted means ± standard errors (SE) and 95% CI. The association between hearing thresholds and candidate risk factors was identified using a multiple linear regression analysis for complex sample design. The complex samples general linear model was then used after adjusting for risk factors that can significantly affect hearing impairment to assess the associations between hearing thresholds and dietary characteristics. Because the nutrients act in concert, a multivariable-adjusted model was used for food and nutrient intake. A *P*-value <0.05 was considered statistically significant.

## 3. Results

### 3.1. General Characteristics

Table 1 shows the study population characteristics and factors associated with hearing impairment. The study population included 5201 adults aged ≥50 years. The age group between 50-year-olds and 60-year-olds was the largest group, accounting for 53% of participants. The weighted means ± SE of the pure-tone average (PTA) at 4FA and HF were 44.0 ± 3.0 dB HL and 61.5 ± 3.0 dB HL, respectively. PTAs at 4FA and HF increased with age, indicating worse hearing (*P* = 0.007 for 4FA and *P* <0.001 for HF). The PTA at HF for men was significantly higher than for women (*P* = 0.029). Lower education level and history of diabetes were also significantly associated with PTAs at 4FA and HF (all *P* <0.05). However, there was no significant difference in PTAs at 4FA and HF between participants exposed to occupational noise and the non-exposed group (*P* = 0.526).

### 3.2. Food Consumption

Table 2 shows the association of food consumption with hearing thresholds after adjusting for age, sex, education level, and history of diabetes. The multivariable-adjusted model revealed that higher intakes of seeds and nuts, fruits, and seaweed were significantly associated with decreased PTAs at 4FA and HF (seeds and nuts: *β* = −0.08, 95% CI = −0.15, −0.01, *P* = 0.013 for 4FA and *β* = −0.07, 95% CI = −0.14, 0.00, *P* = 0.025 for HF; fruits: *β* = −0.01, 95% CI = −0.02, 0.00, *P* < 0.001 for 4FA and *β* = −0.01, 95% CI = −0.02, 0.00, *p* < 0.001 for HF; seaweed: *β* = −0.17, 95% CI = −0.30, 0.04, *P* = 0.001 for 4FA and *β* = −0.16, 95% CI = −0.29, 0.03, *P* = 0.003 for HF).

### 3.3. Nutrient Intake

Table 3 shows the association of macro- and micro-nutrient intake with hearing thresholds after adjusting for age, sex, education level, and history of diabetes. The multivariable-adjusted model found that there was no association between macro-nutrients and PTAs. However, the multivariable-adjusted model for micro-nutrients revealed that higher intake of vitamin A was significantly associated with decreased PTAs at 4FA and HF (*β* = −0.01, 95% CI = −0.01, 0.00, *P* = 0.047 for 4FA and *β* = −0.01, 95% CI = −0.01, 0.00, *p* = 0.024 for HF). The other micro-nutrients were not significantly associated with PTAs.

## 4. Discussion

Our results revealed that higher dietary intakes of seeds and nuts, fruits, seaweed, and vitamin A were positively associated with better PTAs at 4FA and HF in a representative subset of South Korean adults aged ≥50 years old, even after adjusting for risk factors of hearing impairment. The adjusting factors included age, sex, education level, history of diabetes, which were significantly associated with hearing impairment in our study population.

Seeds and nuts, fruits, and seaweed have considered anti-inflammatory food ingredients [38,44,45,46]. There is growing acceptance that inflammation could play a role in ARHL [16,19,32,33]. Studies in a mouse model of ARHL revealed that mature resident macrophages on the basilar membrane display dynamic changes in their morphologies and numbers as age increases, and their changes are related to the phase of hair cell degeneration [33,47]. These findings suggest that the macrophage-mediated immune response is an integral part of the cochlear response to age-related chronic hair cell degeneration. Moreover, experimental data suggest that permeability changes of the blood-labyrinthine barrier, induced by macrophages and macrophage-like melanocytes, may induce the vasospasm of stria vascularis [19,48,49]. Additionally, the other experimental data showed the relationship between age-related immune response and hearing loss caused by the impairment of spiral ganglion cells in the cochlea [50,51]. Together, these results suggest that consuming anti-inflammatory foods could be important for ameliorating ARHL. However, an observational cohort study found that pro-inflammatory foods like sugars and alcohol were associated with ARHL, but not anti-inflammatory foods [38]. Contrary to previous studies, our data found that higher consumption of seeds and nuts, fruits, and seaweed were positively associated with better PTAs at 4FA and HF, but not pro-inflammatory foods.

Seeds and nuts, fruits, and seaweed are also known to contain antioxidant vitamins such as vitamins A, C, and E [44,45,46]. Antioxidants, which inhibit the formation of free radicals, may play a specific role in preventing and treating ARHL [24,27,28,29,38,39,40,52,53,54]. Consistent with previous studies, our data found that higher vitamin A intake was inversely associated with ARHL [38,52,53,54]. Vitamin A, which is required for normal development of the inner ear, comprises several compounds, including retinol, retinal, and several pro-vitamin A carotenoids (most notably β-carotene) [55,56,57]. Antioxidant activity has been reported for vitamin A, as well as for many pro-vitamin A carotenoids [58,59,60,61,62]. Vitamin A and carotenoids were shown to be an effective singlet oxygen and peroxyl radical scavenger [58,59,60]. Thus, vitamin A could scavenge the free radicals produced with aging and may prevent cochlear damage caused by high levels of toxic free radicals. As part of normal cellular homeostasis, free radicals, especially reactive oxygen species (ROS), are continuously generated during aerobic respiration, mostly in mitochondria [27]. Under normal conditions, adequate intracellular ROS levels are essential to regulate many cell-signaling pathways and cellular homeostasis [63], but excessive free radicals cause oxidative stress-induced cell damage as a consequence of imbalances in production of free radicals and endogenous antioxidant systems [27,64]. Specifically, an excess of free radicals in spiral ganglion neurons may play a relevant role in cochlea damage by causing oxidative damage in cells [27,65]. In addition, free radical formation in the stria vascularis causes blood flow reduction in the cochlea and thus, death of the hair cells [27,28]. Subsequent reperfusion of the inner ear further contributes to free radical formation and further cell death [66].

However, retinol, and retinal and retinoic acid are physiologically active forms of vitamin A, and all are toxic in high concentration, meaning that surplus vitamin A must be stored. Depending on the amount of retinol available to the body, pro-vitamin carotenoids can be converted into vitamin A. Therefore, vitamin A intake was expressed in terms of RE in the KNHANES. Both retinol and β-carotene are known to exert antioxidant activities by scavenging free radicals, yet the association between PTAs and retinol and β-carotene intake was not significant in our data. We cannot rule out the possibility that the level of retinol and β-carotene intake was not high, so a significant association could have been observed only in units of weight as RE. In contrast to previous reports, our study found no significant association between PTAs and other dietary antioxidants, such as vitamins B and C [24,67]. Antioxidants may be most effective when working together with other nutrients, but our multivariable model did not include other nutrients, such as vitamin B-12 and E, folate, and magnesium.

Since our study was based on a cross-sectional national survey, there are some limitations that do not explain the causal relationships between the dietary factors and ARHL. Although the fidelity of our data was ensured by using large population data based on the representative sampling of the KNHANES, selection bias remains possible in this study. Because this study was based on a 24 h recall method, some level of recall bias is inevitable. Additionally, 24 h diet recalls might not fully reflect participants’ typical diet. Finally, our results cannot be generalized to other ethnic groups with different dietary habits. To better understand the causal effect of a particular food or nutrient intakes on the development of ARHL, well-conducted intervention studies with dietary factors already identified to be related to ARHL are needed.

## 5. Conclusions

In conclusion, our population-based study suggested that higher intake of seeds and nuts, fruits, seaweed, and vitamin A may decrease the risk of ARHL. Multifactorial nutritional factors are responsible, in part, for ARHL; thus, proper diet counseling could prevent some of the risks and burdens of ARHL.

## Figures and Tables

**Table 1:** Characteristics of participants and factors associated with hearing loss (*N* = 5201).

PTA (dB HL)		4FA	HF
Variables	Values	44.0 ± 3.0 (38.2, 50.0)	61.5 ± 3.0 (55.7, 67.3)
		*P*-Value	*P*-Value
Age, years	60.9 ± 0.2 (61.6, 61.2)	**0.007**	**<0.001**
50–59	2164 (53%)		
60–69	1742 (28%)		
≥70	1295 (19%)		
Sex			
Men	2576 (52%)	0.542	**0.029**
Women	2625 (48%)		
Household income			
Low	1441 (25%)	0.344	0.285
Middle-low	1356 (26%)	0.386	0.322
Middle-high	1169 (24%)	0.969	0.997
High	1235 (25%)		
Education level			
≤Elementary school	2174 (41%)	**0.004**	**0.002**
Middle school	942 (19%)	**0.022**	**0.011**
High school	1370 (27%)	**0.031**	**0.021**
≥College	715 (13%)		
Occupation			
Employed	2865 (61%)	0.720	0.727
Unemployed	2,336 (39%)		
Body mass index (kg/m^2^)	24.1 ± 0.1 (24.0, 24.2)	0.116	0.128
History of hypertension	2057 (37%)	0.500	0.482
History of diabetes	719 (14%)	0.017	0.019
History of noise exposure	698 (15%)	0.433	0.526
Current smoking	906 (21%)	0.491	0.560
Alcohol drinking within past year	4019 (79%)	0.530	0.595

Categorical variables are denoted by unweighted frequencies (weighted percent) and continuous variables are denoted by weighted means ± SE (95% CI). Bold indicates significance (*p* < 0.05). PTA: pure-tone average, 4FA: average for four frequencies (0.5, 1, 2, and 4 kHz), LF: low frequencies (0.5 and 1 kHz), MF: middle frequencies (2 and 3 kHz), HF: high frequencies (4 and 6 kHz).

**Table 2:** Association between food consumption and hearing thresholds.

Food Groups	Values	4FA		HF	
		Estimate	*p* *	Estimate	*p* *
		(95% CI)		(95% CI)	
Cereals and grain products	314.2 ± 2.9 (308.4, 320.0)	−0.02 (−0.06, 0.01)	0.105	−0.02 (−0.05, 0.01)	0.122
Potatoes and starches	36.5 ± 2.2 (32.1, 40.8)	−0.01 (0.04, 0.02)	0.516	−0.01 (0.04, 0.02)	0.533
Sugars and sweets	8.3 ± 0.3 (7.8, 8.9)	−0.20 (−0.66, 0.27)	0.389	−0.21 (−0.67, 0.25)	0.355
Legumes and their products	40.6 ± 1.4 (37.9, 43.4)	0.02 (−0.08, 0.11)	0.719	0.02 (−0.08, 0.11)	0.732
Seeds and nuts	6.1 ± 0.5 (5.1, 7.0)	−0.08 (−0.15, −0.01)	**0.013**	−0.07 (−0.14, 0.00)	**0.025**
Vegetables	355.2 ± 5.4 (344.7, 365.7)	0.01 (−0.01, 0.02)	0.895	0.01 (0.00, 0.02)	0.742
Mushrooms	4.1 ± 0.4 (3.3, 4.9)	0.18 (−0.18, 0.54)	0.317	0.18 (−0.18, 0.53)	0.335
Fruits	198.2 ± 7.5 (183.6, 212.9)	−0.01 (−0.02, 0.00)	**<0.001**	−0.01 (−0.02, 0.00)	**<0.001**
Meats, poultry, and their products	73.3 ± 2.5 (183.6, 212.9)	0.03 (−0.02, 0.09)	0.236	0.03 (−0.02, 0.09)	0.262
Eggs	73.3 ± 2.5 (68.4, 78.2)	−0.05 (−0.18, 0.08)	0.611	−0.05 (−0.18, 0.07)	0.594
Fish and shellfish	15.2 ± 0.6 (14.1, 16.3)	0.04 (−0.05, 0.14)	0.452	0.04 (−0.06, 0.13)	0.481
Seaweed	54.8 ± 1.9 (51.1, 58.5)	−0.17 (−0.30, −0.04)	**0.001**	−0.16 (−0.29, −0.03)	**0.003**
Milk and dairy products	61.3 ± 2.5 (56.3, 66.3)	−0.01 (−0.05, 0.02)	0.508	−0.01 (−0.05, 0.02)	0.505
Fats and oils	6.4 ± 0.2 (6.1, 6.8)	0.16 (−0.75, 1.08)	0.865	0.15 (−0.76, 1.05)	0.896
Beverages	218.0 ± 9.3 (199.7, 236.3)	0.0 (−0.01, 0.02)	0.710	0.0 (−0.01, 0.02)	0.708
Seasonings	33.7 ± 0.7 (32.3, 35.1)	−0.07 (−0.20, 0.05)	0.164	−0.08 (−0.20, 0.04)	0.143

Continuous variables are denoted by weighted means ± SE (95% CI). Bold indicates significance (*p* < 0.05). * Multivariable analysis after adjusting for age, sex, education level, and history of diabetes. 4FA: average for 4 frequencies (0.5, 1, 2, and 4 kHz), HF: high frequencies (4 and 6 kHz).

**Table 3:** Association between nutrient intake and hearing thresholds.

Nutrients	Values	4FA		HF	
		Estimate	*p* *	Estimate	*p* *
		(95% CI)		(95% CI)	
**Macro-nutrients**					
Total energy (kcal/day)	1952.5 ± 15.3 (1922.4, 1982.6)	0.01 (−0.02, 0.04)	0.397	0.01 (−0.02, 0.04)	0.433
Carbohydrate (g/day)	328.2 ± 2.6 (323.0, 333.4)	−0.11 (−0.22, 0.00)	0.060	−0.10 (−0.21, 0.04)	0.072
Protein (g/day)	67.5 ± 0.7 (66.2, 68.8)	0.09 (−0.26, 0.45)	0.605	0.10 (−0.25, 0.45)	0.582
Fat (g/day)	32.9 ± 0.5 (31.9, 33.9)	−0.25 (−0.64, 0.14)	0.205	−0.25 (−0.63, 0.14)	0.208
**Micro-nutrients**					
Crude fiber (g/day)	8.1 ± 0.1 (7.8, 8.4)	−0.29 (−0.82, 0.23)	0.271	−0.26 (0.78, 0.26)	0.323
Ash (g/day)	19.8 ± 0.2 (19.4, 20.3)	0.22 (−0.74, 1.17)	0.658	0.25 (−0.70, 1.20)	0.599
Calcium (mg/day)	505.6 ± 6.7 (492.4, 518.8)	−0.01 (−0.03, 0.02)	0.638	−0.01 (−0.03, 0.02)	0.630
Iron (mg/day)	15.9 ± 0.3 (15.2, 16.5)	−0.10 (−0.25, 0.06)	0.214	−0.10 (−0.25, 0.05)	0.196
Potassium (mg/day)	3090.4 ± 33.1 (3205.4, 3155.4)	0.00 (−0.01, 0.00)	0.458	0.00 (−0.01, 0.00)	0.497
Sodium (mg/day)	4829.1 ± 63.7 (4704.0, 4954.2)	0.0 (0.0, 0.0)	0.750	0.0 (0.0, 0.0)	0.803
Phosphorus (mg/day)	1162.7 ± 9.4 (114.3, 1181.2)	0.00 (−0.02, 0.03)	0.727	0.01 (0.00, 0.02)	0.748
Retinol (μg/day)	75.7 ± 3.9 (68.1, 83.4)	0.01 (0.00, 0.02)	0.279	0.01 (0.00, 0.02)	0.177
β-carotene (μg/day)	4455.5 ± 138.1 (4184.1, 4726.8)	0.0 (0.0, 0.0)	0.102	0.0 (0.0, 0.0)	0.050
Vitamin A (μgRE/day)	836.8 ± 24.6 (788.4, 885.2)	−0.01 (−0.01, 0.00)	**0.047**	−0.01 (−0.01, 0.00)	**0.024**
Thiamin (mg/day)	1.3 ± 0.0 (1.2, 1.3)	−5.61 (−19.39, 8.17)	0.424	−4.77 (−18.27, 8.72)	0.487
Riboflavin (mg/day)	1.1 ± 0.0 (1.1, 1.2)	2.70 (−4.50, 9.90)	0.462	2.32 (−4.75, 9.39)	0.520
Niacin (mg/day)	16.4 ± 0.2 (16.0, 16.7)	0.36 (−0.50, 1.22)	0.414	0.26 (−0.59, 1.10)	0.552
Vitamin C (mg/day)	109.8 ± 1.9 (106.0, 113.5)	0.00 (−0.06, 0.06)	0.974	0.00 (−0.07, 0.06)	0.880

Categorical variables are denoted by unweighted frequencies (weighted percent). Bold indicates significance (*p* < 0.05). * Multivariable analysis after adjusting for age, sex, education level, and history of diabetes. HF: high frequencies (4 and 6 kHz), RE: Retinol Equivalents.

## Data Availability

Data are available from the Korea National Health and Nutrition Examination Survey (KNHANES) Data Access for researchers. Korea Center for Disease Control and Prevention publish annual reports and microdata from KNHANES with survey manuals through the official website of KNHANES (http://knhanes.cdc.go.kr accessed on 28 February 2021), therefore, all KNHANES data are de-identified and available to the public.
